# The significance of petroleum bitumen in ancient Egyptian mummies

**DOI:** 10.1098/rsta.2016.0229

**Published:** 2016-10-28

**Authors:** K. A. Clark, S. Ikram, R. P. Evershed

**Affiliations:** 1 Organic Geochemistry Unit, School of Chemistry, University of Bristol, Cantock's Close, Bristol BS8 1TS, UK; 2 Department of Egyptology, The American University in Cairo, AUC Avenue, PO Box 74, Tagammu 5, New Cairo 11835, Egypt

**Keywords:** mummification, asphalt, quantification, biomarkers, radiocarbon analysis

## Abstract

Mummification was practised in ancient Egypt for more than 3000 years, emerging from initial observations of buried bodies preserved by natural desiccation. The use of organic balms (and other funerary practices) was a later introduction necessitated by more humid burial environments, especially tombs. The dark colour of many mummies led to the assumption that petroleum bitumen (or natural asphalt) was ubiquitous in mummification; however, this has been questioned for more than 100 years. We test this by investigating 91 materials comprising balms, tissues and textiles from 39 mummies dating from *ca* 3200 BC to AD 395. Targeted petroleum bitumen biomarker (steranes and hopanes) analyses by gas chromatography-mass spectrometry selected ion monitoring (GC-MS SIM, *m/z* 217 and 191) showed no detectable bitumen use before the New Kingdom (*ca* 1550–1070 BC). However, bitumen was used in 50% of New Kingdom to Late Period mummies, rising to 87% of Ptolemaic/Roman Period mummies. Quantitative determinations using ^14^C analyses reveal that even at peak use balms were never more than 45% *w/w* bitumen. Critically, the dark colour of balms can be simulated by heating/ageing mixtures of fats, resins and beeswax known to be used in balms. The application of black/dark brown balms to bodies was deliberate after the New Kingdom reflecting changing funerary beliefs and shifts in religious ideology.

This article is part of the themed issue ‘Quantitative mass spectrometry’.

## 1. Introduction

**Figure 1.**
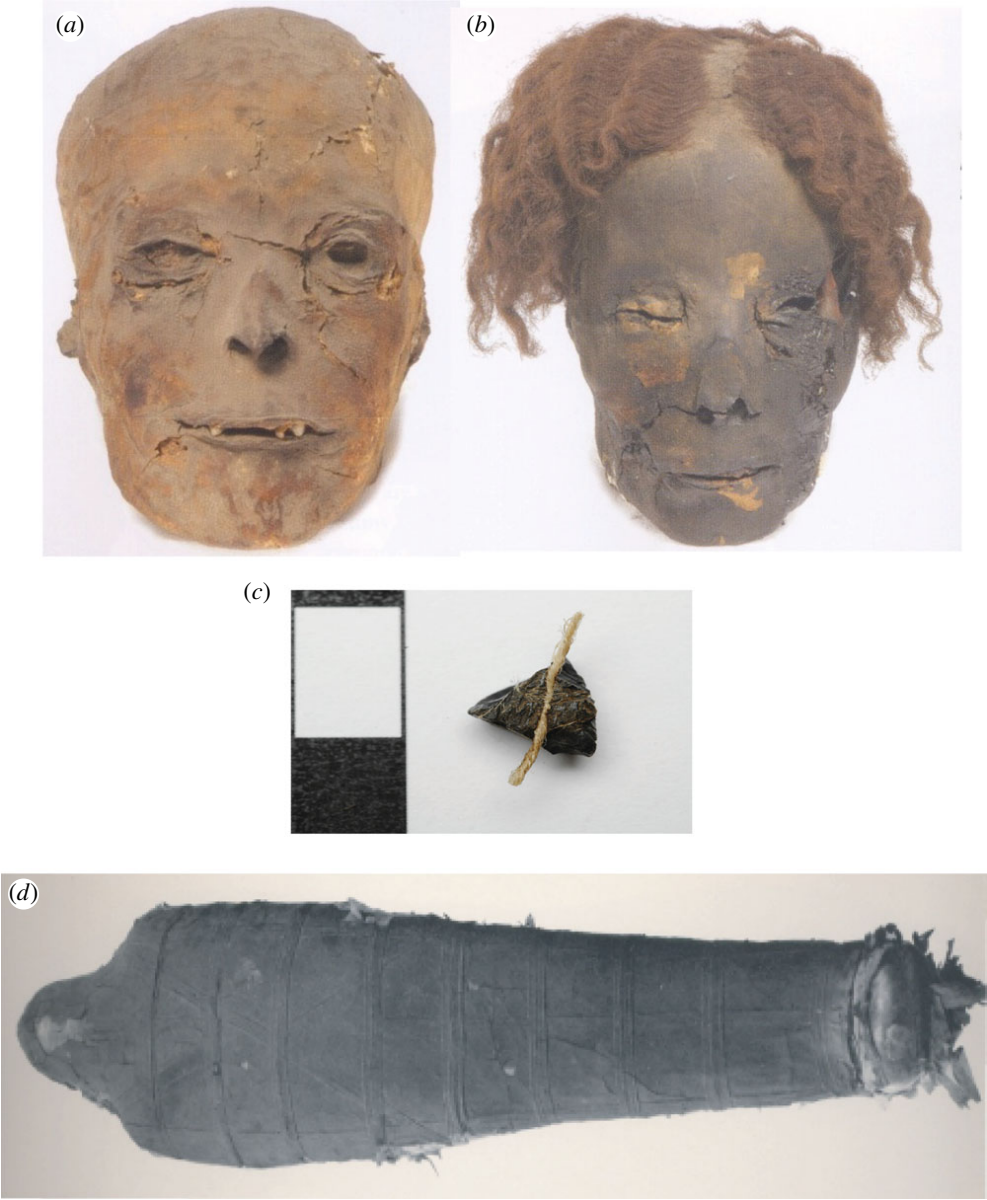
Examples of a typical blackened mummy part (*a*; female adult RMO 41) compared with another mummy (*b*; male adult (RMO 40). (*c*) ‘Resin’ lump with attached thread from the right ankle of female adult (332–30 BC; NMS 1956.352) example of sample used in ^14^C determinations. (*d*) Extensively blackened adult male XXI dynasty mummy (1064–948 BC, BM 6660). (Online version in colour.)

When the Arabs initially encountered mummified bodies, the consistency of the embalming materials and their dark colour led them to believe that the substance was bitumen known to them as *mum*. Hence, the Arabs called the bodies thus prepared ‘*mumia*’, after the black material they thought had been used to make them (figure 1). Both the name for Egyptian preserved bodies (mumia, mummy, momie, mumie, mummia, etc.) and the idea that their preservation was due to bitumen were widely adopted, including by Egyptologists from the nineteenth century onwards [1–6].

Critically, the modern term ‘bitumen’ refers to a specific naturally occurring petroleum product, also known as asphalt, that has lost its volatile hydrocarbon components via biodegradation and/or evaporation, leaving a black, semi-viscous, or even solid, material. However, as bitumen was black, early Egyptologists and more recent researchers, fell into the habit of describing all black mummy balms as ‘bitumen’, when its ubiquity certainly should not be assumed when its presence has only been confirmed in a handful of individuals [1–3,5].

Significantly, there is no consensus among the classical authors as to bitumen use in mummification. Diodorus Siculus, in the first century BC [7], and Pliny [8], in the first century AD, mention bitumen when writing about the Dead Sea. The former mentions that bitumen was sold to the Egyptians for embalming but it is not mentioned when he discusses mummification. Herodotus [9], writing in the fifth century BC, and who of all the classical authors provides the most thorough descriptions of mummification, makes no mention of the use of bitumen during the process, but does describe its use in other contexts completely unrelated to preserving the dead. Strabo [10] reports the sources of bitumen common in the first century BC, and also refers to the substance when writing about the Dead Sea, and states: *The Egyptians use the asphalt for embalming the dead*. Until recently, it was thought that the trade route for the Egyptians to the Dead Sea was only available in Ptolemaic and later times [11], but archaeological discoveries and chemical analyses have revealed molecular evidence for trade during the earlier Chalcolithic and Early Bronze Age periods (3900–2200 BC; [12]). Clearly, therefore, bitumen was available to the Egyptians, but its role in mummification is poorly documented.

The dark or black colour is an unsatisfactory diagnostic for bitumen as many organic materials when heated, aged and/or decayed naturally darken. Thus, it is possible that the dark colour of many of the balms may be ascribed to a variety of non-bituminous sources [13]. It was only in the early twentieth century that some chemists questioned the fact whether all black balms were indeed bitumen [3], and from that time on, with subsequent tests of mummification material it became clear that a variety of materials, including petroleum bitumen, were employed in mummification throughout its over 3300 year history in Egypt [14–27].

Given the uncertainties that exist concerning the importance of bitumen in ancient Egyptian mummification, our aim here was to assess the true historical and quantitative significance of petroleum bitumen in balms in ancient Egyptian mummification across the entire period from 3200 BC to AD 395. To achieve this, we have chemically investigated, using gas chromatography-mass spectrometry (GC-MS) and accelerator mass spectrometry (AMS), the largest collection of mummy balms to be thus analysed in modern times; the collection included 91 samples from 39 mummies (see electronic supplementary material, table S1 for complete list) of tissues, ‘resins’ and bandaging dating from Predynastic to Roman era mummies. GC-MS with selected ion monitoring (SIM) was used to screen all the 91 balms for the presence of bitumen biomarkers, principally hopanes and steranes, allowing the use of bitumen in balms to be tracked chronologically. AMS was then used to determine the concentration of bitumen in a subset of balms. The results obtained (i) confirm the timing of the introduction of bitumen into routine use in mummy balms, and (ii) provide for the first-time accurate quantitative estimates of the proportion of bitumen used in formulating balms.

## 2. Material and methods

### (a) Samples and sample treatments

Samples of tissue, ‘resin’ and bandaging were obtained from mummies from a range of museums (electronic supplementary material, table S1). Samples were taken from various locations on the body and differed widely in visual appearance. Ground samples (*ca* 1–10 mg for pure embalming ‘resins’ and 50–100 mg for tissues and bandaging depending on balm content) were extracted with CHCl_3_/MeOH (2 : 1 *v/v*, 3×) using ultrasonication. The extracts were combined and the solvent removed under a gentle stream of N_2_ at 40°C. Extract yields were determined by weighing. The extracts were separated into ‘acid’ and ‘neutral’ fractions using bonded aminopropyl solid-phase extraction cartridges (100 mg; Varian) with the ‘neutral’ fraction collected by elution with CH_2_Cl_2_/propan-2-ol (2 : 1 *v/v*; 3 ml). The ‘neutral’ fraction was further separated using a column of activated silica gel following elution with hexane to give a hydrocarbon fraction.

### (b) Gas chromatography-mass spectrometry selected ion monitoring

All the hydrocarbon factions were submitted to GC-MS using a Finnigan Trace instrument (Finnigan MAT GmbH, Bremen, Germany) equipped with an on-column injector. The mass spectrometer was set to scan in the range of *m/z* 50–700 in a total time of 0.6 s. For SIM, the mass spectrometer was set to monitor *m/z* 191 (hopanes and other triterpanes) and 217 (steranes) [20,23]. The GC column was a CPSIL-5 (60 m × 0.32 mm × 0.1 µm) and the operating conditions were 50–130°C at 20°C min^−1^, to 300°C (held 30 min) at 40°C min^−1^. He was used as carrier gas, the electron emission current was 300 µA, the ion source temperature was 170°C and the GC-MS interface was maintained at 350°C. The electron ionization potential was 70 eV.

Each hydrocarbon fraction was run twice, first without internal standards. Only those bitumen hydrocarbon fractions displaying detectable peaks (*s/n* > 5 : 1) at appropriate retention times in the initial sterane and hopane analyses were submitted to quantitative analysis. The integral of all the peak areas in the *m/z* 217 and 191 mass chromatograms provided the basis of the quantification based on co-injected standards. Quantification of the sterane and hopane biomarkers was achieved through electronic integration of the peak areas and comparison with the area of co-injected standards; 5α-cholestane was selected for the steranes and hop-21-ene for the hopanes as these have similar fragmentation patterns and ion yields to the group of biomarkers being quantified. A calibration curve was determined prior to analysis to aid the choice of a suitable standard concentration. The limit of detection of these standards was found to be 0.05 ng of hopane and 0.005 ng of sterane injected.

The concentration of biomarkers in the extracted mummy balm was then calculated using these standards and using the weight of the aliquot of the total lipid extract fractionated into the saturated hydrocarbon fraction and the volume the saturated hydrocarbon fraction was dissolved in for analysis. The integral of all the peak areas in the *m/z* 191 and 217 mass chromatograms provided the basis of the quantification. The concentrations of hopanes and steranes were calculated as follows:
2.1
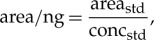

2.2
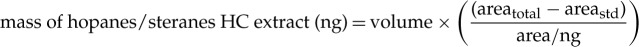

2.3
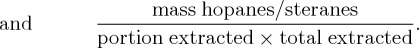


The difference in the way the sterane and hopane concentrations are determined is due to the way the internal standards are used. The area of the added hop-21-ene is readily determined as it does not co-elute with any component of the bitumen. However, the area of the co-injected 5α-cholestane must take account of the cholestane endogenous to the archaeological or reference bitumen. This is overcome in the standard addition method by using the difference in peak area with and without the co-injected 5α-cholestane standard. In all calculations, the reasonable assumption is made of similar response factors between the analytes and co-injected standards.

The errors associated with determination of the biomarkers using the co-injection method are principally associated with calculation of the concentration of the standard, the measurement of the aliquot of both the standard and sample for co-injection and the initial weighing of the sample, insoluble residue and the aliquot used for fractionation. The various points at which the sample was weighed have an associated error of ±0.0005 g, which for a sample size of 50 mg accounts for an error of approximately 1%. As each sample was weighed three times (initial sample, insoluble residue and aliquot for fractionation), this gives an overall error of 2% (≈√(1^2^ + 1^2^ + 1^2^)). The error in the concentration of the standard is 1% for the weighing of the standard and 10% for the volume, giving an overall error of 10%. Finally, the error of the co-injection is also calculated to be 10%. These errors combined give a total error for the determination of the biomarker concentration in bitumen of approximately 15%. Given the latter biomarker concentrations are reported in electronic supplementary material, table S1 as semi-quantitative concentration ranges rather than precise concentrations: + = 0.01–0.1 µg g^−1^ and 0.1–1 µg g^−1^; ++ = 0.1–1 µg g^−1^ and 1–10 µg g^−1^; +++ = 1–10 µg g^−1^ and 10–100 µg g^−1^; ++++ = 10–100 µg g^−1^ and 100 to greater than 1000 µg g^−1^, for steranes and hopanes, respectively.

### (c) Radiocarbon analyses by accelerator mass spectrometry

A subset of samples of ‘resin’ and bandaging (purified cellulose [28]) were analysed at the Oxford Radiocarbon Accelerator Unit (ORAU, UK) using a continuous-flow CHN analyser (Europa-ANCA) fitted with a CO_2_ collection facility to provide CO_2_ as the target material for gas source AMS [29]. From AMS analysis, a value for the ^14^C content can be derived, expressed as % modern ^14^C. Isotopic fractionation effects are accounted for by normalizing the measurements to the common δ^13^C value of −25‰ and adding or subtracting 8.2 ^14^C years for each 1‰ difference. The radiocarbon age can be expressed as a radiocarbon age (in years BP) using the following expression:
2.4
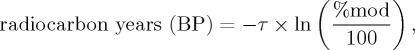

where *τ* is the Libby mean-life (8033 years) and %mod is the percentage of ^14^C remaining relative to modern levels (i.e. AD 1950). This corrected age is then be calibrated against the [30] calibration curve using the OxCal v. 3.9 program [31] to provide a calendar date range.

The radiocarbon ages of balm and the textile samples (e.g. figure 1) were used to determine bitumen content on the basis of the differences in their dates, *Δ* radiocarbon years. The presence of 1% radiocarbon ‘dead’ carbon shifts the true age by 80 years (see equations (2.5) and (2.6); [32]). Contamination by radiocarbon-dead carbon will affect the measured radioactivity of the balm according to the expression
2.5
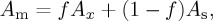

where *A*_m_ is the measured activity, *A_x_* the activity of the contaminant (i.e. petroleum bitumen), *A*_s_ the activity of the true sample and *f* the fraction of the contamination. Conversion of measured activity to time is achieved using the following standard radioactivity equation (substituting *A_x_* and *A*_s_):
2.6
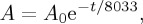

where 8033 years is Libby's mean-life and *A*_0_ is the modern activity.

Where contamination is by infinitely old carbon, *A_x_* = 0, so, using the above equations, it can be shown that 1% of infinite age (dead) carbon adds *ca* 80 years to the apparent age of the sample. Rearrangement of the radiocarbon equations gives the percentage of ‘dead’ carbon present from the difference in radiocarbon ages as the following equation:
2.7
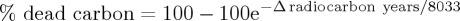


and
2.8
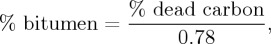

where Δ radiocarbon years = radiocarbon age (resin/tissue)—radiocarbon age (textile).

Bitumen from the Dead Sea contains 78% carbon [12], which can be used to convert % dead carbon to % bitumen. The difference, Δ radiocarbon years, was calculated using the convolution of the two functions (equation (2.9)) which also gives the associated error [31]:
2.9
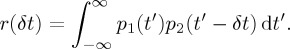

The convolution effectively ‘blends’ one function (*p*_1_ or the radiocarbon age of the bandage) with another function (*p*_2_ or the radiocarbon age of the ‘resin’) giving the distribution of the difference in radiocarbon age.

## 3. Results and discussion

The investigation proceeded to two stages. Initially, extracts of mummy balms, textiles and tissues were screened for the presence of diagnostic hopane (and other triterpane) and sterane petroleum biomarkers using GC-MS with SIM. The results of this screening phase were used to map the use of petroleum bitumen through time and also to identify a subset of samples for subsequent radiocarbon analysis to quantify the petroleum bitumen concentration in mummy balms.

### (a) Screening mummy balms for petroleum biomarkers

**Figure 2.**
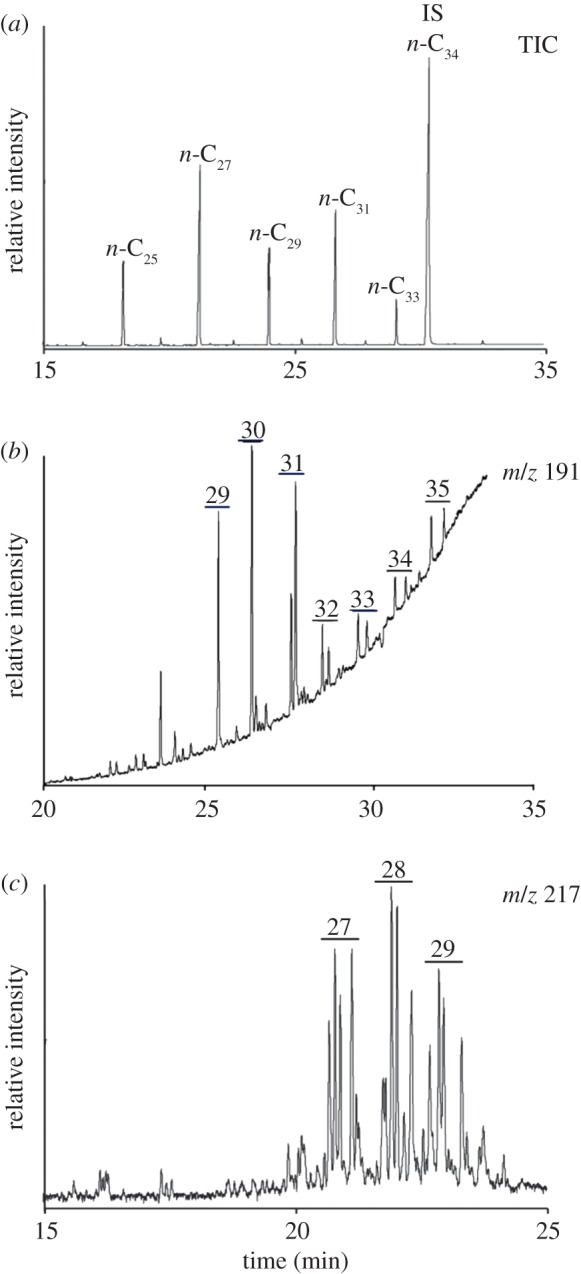
Total ion current (*a*; TIC), obtained by GC-MS and (*b*) *m/z/z* 191 and (*c*) 217 chromatograms, determined by GC-MS with SIM, of saturated hydrocarbon fraction of ‘resin’ attached to a linen thread from the right ankle of a female adult (332–30 BC; NMS 1956.352). In the TIC, the numbers on the peaks correspond to the carbon numbers of the major *n*-alkanes, which derive predominantly from beeswax. In the GC-MS SIM *m/z* 191 and 217 mass chromatograms, the horizontal bars correspond to the retention time windows within which the major hopanes (and other triterpane biomarkers, i.e. oleanane, small peak eluting just before the C_30_ hopane and gammacerane, small peak eluting between the C_31_ and C_32_ hopanes) and sterane biomarkers elute. The numbers denote the carbon numbers of the components eluting within those ranges. The multiple peaks within the carbon number group under each horizontal bar correspond to isomeric mixtures produced during the petroleum formation process. Further explanation of the biomarker compositions are given by Connan [23]. The *m/z* 191 ion for hopanes is formed by EI cleavage of the C-ring with charge retention on the A + B ring containing fragment, while the *m/z* 217 ion for steranes is formed by D-ring cleavage with charge retention on the A + B + C ring fragment (see Fig. 2 in reference [20]). The rising baseline in the *m/z* 191 mass chromatogram arises from the presence of the ion in the column bleed accentuated by the low concentration of hopanes.

All the balms were initially screened by GC-MS to determine the major fat/oil, di-/triterpenoid resin and beeswax components of the balms [24]. Since the biomarkers for petroleum bitumen are known to be present in trace concentrations, extracts were fractionated to yield saturated hydrocarbon fractions (figure 2*a*) that were analysed by GC-MS SIM (figure 2*b,c*; *m/z* 191 and 217) for the targeted analysis of bitumen sterane and hopane biomarkers at high sensitivity (results are summarized in electronic supplementary material, table S1). The analytical procedure was validated by analysing reference bitumens including Dead Sea, Gebel Zeit and Abu Durba, which gave analogous SIM chromatograms [24] to those published in Harrell & Lewan [20]. Steranes concentrations were always lower than hopanes (and other triterpanes). The difficulties in detecting steranes and hopanes in mummy balms indicate that the bitumen biomarkers are present at low concentrations. Quantification of biomarkers was performed where they were deemed to be present in sufficiently high concentrations (electronic supplementary material, table S1). The results show that in the majority of mummy balms the concentration of biomarkers range between approximately 10 µg g^−1^ and 500 µg g^−1^; the highest concentration detected was from the tissues of a female Greek mummy (MTB 7700/4963) where the concentration of hopanes was approximately 1500 µg g^−1^. In all cases, the concentrations of steranes and hopanes are considerably lower (by several orders of magnitude) than the concentrations of lipids from fats/oils, beeswax and resins found in the balm, which are found in typically mg g^−1^ concentrations. Where detected the majority of the bitumens identified can be attributed to the previously recognized Dead Sea source [18–21,24].

**Figure 3.**
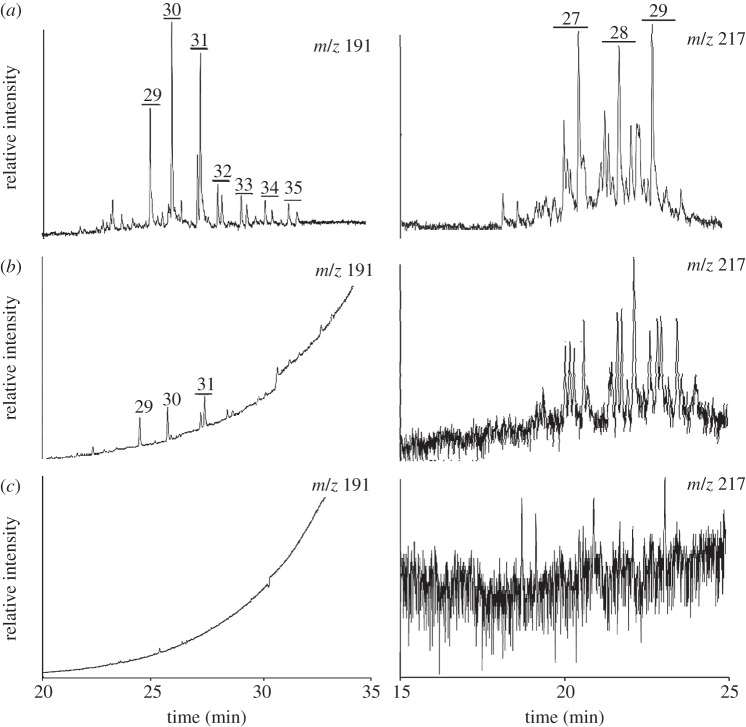
GC-MS SIM *m/z* 191 and 217 mass chromatograms of saturated hydrocarbon fractions of: (*a*) ‘resin’-coated bandages of young male adult (*ca* 332–330 BC; BRI Ha7385), example of a balm characterized as +++ in electronic supplementary material, table S1; (*b*) bandaging from the left hand of a Third Intermediate Period male adult (*ca* 1064–927 BC; MTB G44), example of a balm characterized as + in electronic supplementary material, table S1 and (*c*) ‘resin’/tissue/bandaging from XXII Dynasty male adult Khnumnakht (*ca* 1994–1781 BC; MAN 21471), example of a ‘balm’ characterised as none detected (Nd; electronic supplementary material, table S1). The horizontal bars in the mass chromatograms correspond to the retention time windows within which the major hopane and sterane components elute. The numbers over the bars denote the carbon numbers of the major hopane and sterane components eluting in those ranges. See Material and methods and caption to figure 2 for further details.

Significantly, none of the mummies dating before *ca* 1000 BC contained detectable bitumen biomarkers. An example of one of these early mummies was the male adult Khumnnakht (Middle Kingdom, *ca* 1985–1795 BC) previously shown to comprise mainly fat/oil [22,33]; GC/MS with SIM indicated no detectable steranes or hopanes (figure 3*c*). By contrast, many mummy balms from later periods of Egyptian history exhibited evidence for the presence of steranes and hopanes (figure 3*a,b*; electronic supplementary material, table S1). Their occurrence was most common in mummies from the Ptolemaic to Roman Periods (332 BC on), rather than from those of the Third Intermediate (*ca* 1064–525 BC) and Late Periods (*ca* 525–332 BC). However, even in the mummy balms deemed to contain bitumen, the steranes and hopanes were present at low concentrations.

### (b) The chronology of bitumen use on mummy balms

**Figure 4.**
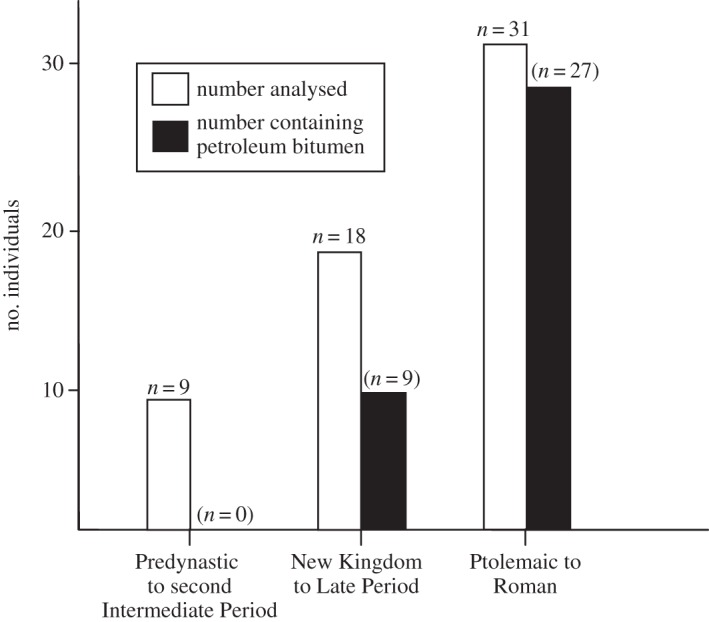
Histogram showing increasing abundance of bitumen in mummy balms from the Predynastic to Roman period. Includes findings of this study and published work [14–16,18,19,22–24,27,34–40].

Figure 4 integrates the results of all mummy balms and tissues analysed for sterane and hopanes characteristic of bitumen. The earliest evidence obtained herein for detectable bitumen was obtained from the Glasgow male mummy (MTB G44), which dates to the Early Third Intermediate Period (*ca* 1064–927 BC; figure 3*b*). An increase in bitumen use is apparent during the Third Intermediate Period, with the peak of use reached in Ptolemaic and Roman times (figure 4; electronic supplementary material, table S1). From the Third Intermediate Period onward, there appears to be a general increase in mummification of individuals, which peaked in the Graeco-Roman era when mummification became even more common across social classes and age groups [5].

It was notable that a number of extremely black mummies lacked detectable bitumen biomarkers, notably the Roman era mummy with the folded arms (TUR Pravv 540), and the XXI dynasty mummy (BM 6660, Third Intermediate Period; figure 1). Both balms were very black and visually might easily be interpreted as being bitumen or containing a significant concentration of bitumen. Interestingly, the mummy with the folded arms (TUR Pravv 540) has been dated to the end of the Roman period and might have been expected to contain bitumen. A number of other mummies dating to the Ptolemaic and Roman periods similarly lacked detectable bitumen biomarkers, such as the male adult with the prosthetic hand (DUR 1999.31.1) and the ‘resin’ from the head of a female adult (RMO 41). These results clearly indicate that the use of bitumen was a complex activity, and that the widespread use of true bitumen was a later introduction. Clearly, the black colour of mummies is an unreliable indicator of the presence of bitumen.

### (c) Determination bitumen concentration in mummy balms

A further important question concerning the importance of bitumen in mummy balms is the proportion of bitumen used to prepare organic balms relative to the other ingredients, such as fat/oil, beeswax and resin [16–18,22,24]. One method of determining the concentrations of bitumen that was used in the past is based on the concentrations of the biomarker components of balms relatively to the source bitumen [15,23]. However, the results obtained using this approach are affected by the natural variability in concentrations of biomarkers in the sources due to varied diagenetic histories, together with possible mixing of bitumen from different sources during the preparation of balms. In view of the latter, we adopted a radiocarbon approach reasoning that since bitumen is of geological age it would be radiocarbon ‘dead’. Thus, the ^14^C content would be negligible, and the presence of any bituminous material in the balm would dilute the ^14^C present in the balm, thereby causing a shift in radiocarbon date towards older ages [41,42]. By comparing this date with the date from other materials from the same mummy, contemporaneous with the body and free of bitumen, it is possible to apportion the concentration of bitumen present in the balm. Hence, a subset of the collection of mummy balms was chosen according to the following criteria: (i) they had well established dates based on archaeological/stylistic/contextual/typological criteria; (ii) they covered a wide range of dates, which did not fall in flat areas of the radiocarbon calibration curve; (iii) samples of bandaging and balm were available from the same mummy; and (iv) variable bitumen concentrations were suggested, based on bitumen biomarker concentrations ranging from mummies with no bitumen, barely detectable bitumen biomarkers to those with readily detectable biomarkers.

**Figure 5.**
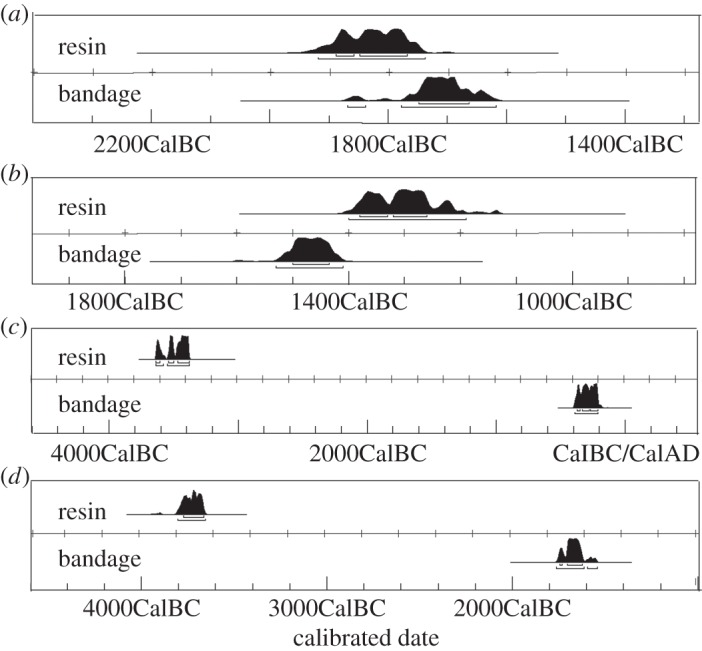
Calibrated radiocarbon dates for textile bandages and balms from (*a*) male adult, Khnumnakht (MAN 21471), (*b*) male adult (Glasgow; MTB G6), (*c*) female adult (NMS 1956.352) and (*d*) male adult (BRI Ha7385). See table 1 and text for further details.

The results of the radiocarbon analyses of the balms and bandaging ‘pairs' are shown in figure 5 and table 1 giving the radiocarbon age, the calibrated age, the difference, Δ radiocarbon years and the percentage of dead carbon (attributed to the presence of bitumen) that would cause the observed differences in the dates. The results from Khumnakht (MAN 21471) show a small negative difference between the dates from the bandaging and the ‘resin’ (table 1) corresponding to the presence of 0–3% of ‘dead’ carbon in the bandaging. As cellulose was purified from the bandaging, the possibility of contamination by bitumen was eliminated and the percentage of dead carbon must be zero. The ‘resin’ from the Glasgow male (MAN G6) also shows a small difference in the radiocarbon age between the bandaging and the ‘resin’ of 40 and 310 years (table 1). This difference in age corresponds between 0.5% and 4% of ‘dead’ carbon and, therefore, a maximum of only 0.6–5% of this balm comprised bitumen. Given the blackened nature of this mummy, which would normally be attributed to the presence of bitumen in the balm, the low fraction of ‘dead’ carbon suggests that the blackened nature of this mummy is due to other factors. Components identified in the latter balm included fatty acids originating from the application of a fat or oil, wax esters from beeswax and diterpenoids from coniferous resin [24]. Hence, the black colour of the mummy must result largely through their darkening during balm production and use or through ageing. Experiments aimed at replicating ancient Egyptian balms have shown that when resins are melted with oils very dark blackened coatings are indeed produced (S. Ikram 2012 and 2014, unpublished data).

**Table 1.** Results of AMS radiocarbon analyses performed on textiles and balms to estimate petroleum bitumen content of balms.

mummy	museum number	laboratory reference number	sample	steranes and triterpanes present^a^	conventional ^14^C age (±)	calibrated age using Oxcal, 2*σ* ranges	difference, Δ/years (resin-bandage)	% of dead carbon	% of bitumen^b^
male adult, Khumnakht	MAN 21471	OxA-14962	bandaging	Nd	3511 (31)	1920–1740 BC (91.2%)			
		OxA-V-2140–10	‘resin’/tissue	Nd	3411 (32)	1780–1620 BC	10–250^c^	0	0
male adult	MTB G6 Glasgow	OxA-14964	bandaging	+	3032 (31)	1400–1190 BC			
		OxA-V-2141–18	‘resin’ attached to bandaging	+	3200 (33)	1530–1410 BC	40–310	0.5–4	0.6–5
female adult	NMS A.1956.352	OxA-14933	linen thread	+++	2211 (30)	380–200 BC			
		OxA-V-2141–20	‘resin lump’ attached to linen thread	+++	4699 (35)	3790–3640 BC	3020–3400	31–35	39–45
male adult	BRI Ha7385	OxA-14934	bandaging	+++	3366 (31)	1750–1600 BC			
		OxA-V-2141–21	resin attached to bandaging	++++	4939 (37)	3630–3570 BC (18.1%)	1920–2200	21–24	27–30
						3540–3370 BC (77.3%)			

^a^Nd, bitumen not detected, +, 0.01–0.1 µg g^−1^ and 0.1–1 µg g^−1^, +++, 1–10 µg g^−1^ and 10–100 µg g^−1^, ++++, 10–100 µg g^−1^ and 100–greater than 1000 µg g^−1^, for steranes and hopanes, respectively. See also §2(b) in ‘Material and methods’ above and electronic supplementary material, table S1 for sterane and hopane data on the other mummies investigated.

^b^Calculated using the %C Dead Sea bitumen (78%) [12].

^c^The radiocarbon date of the bandaging is older than that of the ‘resin’/tissue and, therefore, the difference calculation is included here for completeness and could be considered as zero.

The findings from the Ptolemaic mummies show considerably greater differences between the radiocarbon ages obtained from the resin and bandaging (table 1). The differences between the dates for ‘resin’ and bandage from the male mummy (BRI Ha7385) is 1920–2200 years, which corresponds to the presence between 21% and 24% of radiocarbon ‘dead’ carbon (27–30% *w/w* bitumen). The difference between the ‘resin’ and bandaging from the female mummy (NMS A.1956.352) is calculated as 3020–3400 years, showing that the presence of 31–35% ‘dead’ carbon, i.e. 39–45% *w/w* bitumen. Given the blackened nature of both of these balms, and the high portion of ‘dead’ carbon it is, therefore, appropriate that these balms are described as bituminous, although interestingly neither are wholly composed of bitumen. We performed analogous calculations for the three Graeco-Roman mummies studied by Aufderheide *et al.* ([41]; electronic supplementary material, table S2), which revealed maximum bitumen concentrations of 19% *w/w*. The differences in radiocarbon ages observed between ‘resins’ and tissues/textiles are consistent with other mummies from the site that have been shown to contain bitumen based on sterane and hopane biomarker analyses [18].

## 4. Conclusion

It has been demonstrated that for the first 2000 years in which mummification was practised prior to the New Kingdom petroleum bitumen (or natural asphalt) was not used in embalming as a general practice. The earliest evidence for the presence of bitumen in a mummy balm derives from a single individual dating to the end of the New Kingdom (1250–1050 BC; [18]). The use of bitumen in balms becomes more prevalent during the Third Intermediate Period, *ca* 750 BC and was extensively used during the Ptolemaic and Roman periods. Radiocarbon analyses have shown that even when present, balms were likely never wholly composed of bitumen. This might reflect its initial rarity, or the belief that some of the traditional materials had to be used if the mummification were to be efficacious.

Although the use of bitumen became widespread in later periods, it was not ubiquitous, as confirmed by sterane and hopane biomarker analyses of mummy balms from these periods. The increase in its use is attributable to a variety of factors, some practical, e.g. antimicrobial properties, as with other components of balms [22,43], and others cultural. It probably provided a simpler and speedier means of mummification—many Graeco-Roman mummies lack the excerebration and in some cases evisceration that were commoner in earlier periods [6,36]—allowing for the embalming of larger numbers of people, across social classes and age groups. Additionally, the various sources likely became increasingly accessible as trade routes opened up and the control of the whole area was in Roman hands.

Another explanation for the introduction of bitumen during the Late and Graeco-Roman Periods might be due to a shift in funerary beliefs that involved colouring the body black. The symbolism associated with the colour black is significant: black was associated with the colour of the rich, fertile silt deposited by the annual Nile flood, a symbol of regeneration, rebirth and resurrection, and a colour, together with green, attached to Osiris, god of the dead, lord of the afterlife [5,42,44], and master of resurrection. By darkening the deceased's body during the final phases of mummification so that it became black, he or she was literally transformed into Osiris (see discussion in [7]), living eternally.

Bitumen itself seems to have been regarded as a commodity associated with sacredness and divinity. The ancient Egyptian word for bitumen is usually translated as *mnnn*, which has parallels with *mny* [45]. Other texts mention ‘*mny* on the flesh of the gods’ [46] and *mnît* mixed with *îhmt* (an unknown substance) to prepare the ointment, *mrhet*, for application to the limbs of the god Amun [47], also a fecundity deity, especially in his guise as Amun-Min/Kamutef [48], who was also shown with black flesh. A recipe from the temple of Edfu lists *mnn* as an ingredient of *aat-netrjret* translated as ‘divine stone’, which was applied to images of the ithyphallic fertility god Min, who himself was often described as black like *mn* [49,50], and was also associated with aspects of Osiris. Thus, based on the results of this study, it would seem that both practical and theological associations with bitumen are responsible for the increase in its use, and of dark coloured balms generally, in the latest periods of Egyptian history, as it democratized death and the transformation of the deceased into Osiris [5,51].

## Supplementary Material

ESM Table 1 and Table 2
