# Supplementary material for: The significance of petroleum bitumen in ancient Egyptian mummies
Source: Philos Trans A Math Phys Eng Sci. 2016 Oct 28;374(2079):20160229. doi: 10.1098/rsta.2016.0229 (PMC5031647; doi:10.1098/rsta.2016.0229)
Supplement: ESM Table 1 and Table 2 [file rsta20160229supp1.docx]

**ESM Table 1** Nature, origin and dates of mummy balms together with the occurrence and concentrations of sterane and triterpane biomarkers Modified from Clark [1].

| Mummy | Museum number^a^ | Date | Provenance | Sample type and location | Triterpanes | Steranes |
| --- | --- | --- | --- | --- | --- | --- |
| Adult | TUR  Drawer 528 | 3200 BC | Gebelein | Light tissue | Nd^c^ | Nd |
|  |  |  |  | Light bone | Nd | Nd |
| Female adult | TUR  Drawer 520 | 3200 BC | Gebelein | Bandage | Nd | Nd |
|  |  |  |  | Tissue from sole of right foot | Nd | Nd |
| Adult | TUR  Drawer 522 | 3200 BC | Gebelein | Bandaging from lower leg | Nd | Nd |
|  |  |  |  | Tissue from lower leg | Nd | Nd |
| Female adult | TUR  Drawer 517 | 3200 BC | Gebelein | Tissue from skull | Nd | Nd |
| Adult | TUR  Drawer 535 | 3200 BC | Gebelein | Bandaging from top of right hand | Nd | Nd |
|  |  |  |  | Tissue from palm | Nd | Nd |
| Female adult with dress | TUR | 2410-2195 BC | U^b^ | Tissue from left frontal/parietal area | Nd | Nd |
|  |  |  |  | Tissue from right leg | Nd | Nd |
|  |  |  |  | Tissue from right temporal area | Nd | Nd |
|  |  |  |  | Tissue inner side right leg | Nd | Nd |
|  |  |  |  | Tissue from inner sided of right forearm | Nd | Nd |
|  |  |  |  | Bandages on torso | Nd | Nd |
|  |  |  |  | Tissue from right forearm | Nd | Nd |
| Male adult, Kuhmnakht | MAN 21471 | 1985-1795 BC | Rifeh | Muscle tissue | Nd | Nd |
| Female adult | NMS 1909.527 | 1650 BC | Qurna | ‘Resinous’ material from bottom of coffin | Nd | Nd |
|  |  |  |  | ‘Resin’ impregnated tissue | Nd | Nd |
|  |  |  |  | ‘Polymerised’ fat in front and middle | Nd | Nd |
|  |  |  |  | Tissue fragment | Nd | Nd |
|  |  |  |  | Stained bandaging | Nd | Nd |
|  |  |  |  | Stained bandaging from cloth doubled under body | Nd | Nd |
| Child (Qurna) | NMS 1909.527 | 1650 BC | Qurna | Stained bandaging | Nd | Nd |
| Head | LIV 1953.72 | 1550-1069 BC | Thebes | Bandaging | Nd | Nd |
| Male adult head, Khonsuhotep | RMO 33 | 1200-1000 BC | Thebes | Tissue/’resin’/bandage fragment and hair | Nd | Nd |
| Male adult,  Djed-Khons-Ankh | BRI H5074 | 1186-656 BC | U | Tissue from left hand side of chest | Nd | Nd |
|  |  |  |  | Bandaging from feet | Nd | Nd |
| Male adult,  Horemkenesi | BRI Ha7386 | 1069-945 BC | Deir el Bahri | ‘Resinous material from left hand side of spine | Nd | Nd |
|  |  |  |  | Bandage from left ankle | Nd | Nd |
| Male adult | BM 6660 | 1064-948 BC | U | Blackened ‘resin’ from stomach area | +^d^ | + |
| Male adult (Glasgow) | MTB G6 | 1064-927 BC | U | Bandage from the back of left hand | + | + |
|  | MTB G44 |  |  | Bandage package-bandage | +++ | ++++ |
| Cornell mummy resin (Penpi) | MTB 5681 | 897-715 BC | U | ‘Resin’ | Nd | Nd |
| Female adult,  Ta Sedgemet | NZ | 850-575 BC | U | Embalming resin from head | Nd | Nd |
| Male child | BRI H6140 | 747-665 BC | U | Tissue from right ankle | Nd | Nd |
| Child (BRI) | BRI Ha7563 | 727-30 BC | U | Bandaging from left hip | ++++ | ++ |
| Male adult,  Bes-en-Mut | MTB 528/1 | 700 BC | Akhmim | Tissue from right foot | + | + |
|  |  |  |  | ‘Resin’ | ++++ | +++ |
|  |  |  |  | Burnt vertebrae | ++++ | +++ |
| Male adult,  Peadamun Impuwer | LIV 1953.72 | 664-404 BC | Thebes | ‘Resin’ top of cranium | ++++ | + |
| Female adult,  Pa-Nesit-Tawy | MTB 528/SLA50.1928 | 650 BC | U | Package | + | + |
|  |  |  |  | Bandage | Nd | Nd |
| Female adult (Greek) | MTB 4158/3347 | 332-30 BC | U | Tissue and bandage | ++++ | +++ |
| Head | MAN 7700/5275 | 332-30 BC | U | Bandage/tissue under left hand side of jaw bone | Nd | Nd |
| Male adult | BRI Ha7385 | 332-30 BC | U | ‘Resin’ coated outer bandages | +++ | ++++ |
| Female adult right foot | BRI H7212 | 332-30 BC | Thebes | Tissue from ankle | + | + |
| Right foot | BRI H5543 | 332 BC-395 AD | U | Bandaging from ankle | + | + |
| Female adult | NMS 1956.352 | 332-30 BC | Thebes | ‘Resinous’ material from amulet on neck | ++++ | + |
|  |  |  |  | ‘Resin’ attached to thread right ankle | +++ | +++ |
|  |  |  |  | Stained bandaging from right hand side of neck | Nd | Nd |
| Male adult with prosthetic hand | DUR 1999.31.1 | 332 BC-395 AD | Luxor | ‘Resin coated outer bandages | Nd | Nd |
| Male adult Djehor, | BM 29776 | 332-30 BC | Akhmim | ‘Resin” coated bandages from left shoulder | +++ | ++ |
| Adult | BM 29783 | 332-30 BC | Akhmim | ‘Resin’ coated bandages from left hand side of shoulder/neck | +++ | +++ |
| Male adult With folded arms | TUR Pravv 540 | 100 BC-395 AD | Assuit | Stained bandaging from leg | Nd | Nd |
|  |  |  |  | ‘Resin’ on stomach | Nd | Nd |
|  |  |  |  | Pale bandaging | Nd | Nd |
| Male adult | NMS 1911.2101 | 30 BC-395 AD | Hawara | ‘Resin’-soaked outer wrapping below right scapula | +++ | +++ |
| Head of a female child | RMO 34 | 30 BC-395 AD | U | Tissue inside neck | ++++ | ++++ |
| Head of a female adult | RMO 35 | 30 BC-395 AD | Saqqara | Bone from left hand side of jaw bone | ++++ | ++ |
| Head of a male adult | RMO 39 | 30 BC-395 AD | U | Tissue/’resin’ | ++ | +++ |
| Head of a female adult | RMO 41 | 30 BC-395 AD | Thebes | Tissue/’resin’ | Nd | Nd |
|  |  |  |  | ‘Resin’ on hair | Nd | Nd |
| Head of a female adult | RMO 44 | 30 BC-395 AD | U | Tissue/’resin’ | +++ | ++++ |
|  |  |  |  | Tissue from neck | ++ | ++ |
| Head of a male adult | RMO 47 | 30 BC-395 AD | U | Tissue | +++ | +++ |

^a^ BM = British Museum; BRI = Bristol City Museum and Art Gallery; DUR = Durham Oriental Museum; LIV = Liverpool Museum; MAN = Manchester Museum; MTB = Manchester Tissue Bank; NMS = National Museum of Scotland; NOR = Norwich Castle Museum; NZ = Auckland War Memorial Museum, New Zealand; RMO = Rijksmuseum van Oudheden, Leiden, The Netherlands; TUR = Museum of Ethnography and Archaeology, Turin, Italy.

^b^U = unknown; ^c^Nd = not detected; ^d^Concentration ranges: + = 0.01-0.1 μg g^-1^ and 0.1-1 μg g^-1^; ++ = 0.1-1 μg g^-1^ and 1-10 μg g^-1^; +++ = 1-10 μg g^-1^ and 10-100 μg g^-1^; ++++ = 10-100 μg g^-1^ and 100->1000 μg g^-1^, for steranes and hopanes, respectively.

ESM Table 2

Difference in radiocarbon dates of textiles and balms from Aufderheide et al. [2] with estimates of bitumen contents of balms.

| *Mummy no.* | *Specimen* | *R/A and laboratory^a^* | *Conventional ^14^C age (±)* | *Calibrated age using Oxcal, 2σ ranges* | *Difference Δ/years*  *(resin-textile or textile-tissue)* | *Dead carbon (%)* | *Bitumen (%)^b^* |
| --- | --- | --- | --- | --- | --- | --- | --- |
| 5 | Resin | R  Geochron | 2225 (105) | 550 BC-50AD (94%) | 10-900 | 0-11 | 0-14 |
| 5 | Muscle | R  Geochron | 1830 (60) | 50-350 AD |  |  |  |
| 7 | Resin | A  Beta analytical | 2580 (50) | 840-520 BC | 530-940 | 6-11 | 7-14 |
| 7 | Textile | A  Beta analytical | 1950 (40) | 50 BC-140 AD |  |  |  |
| 15 | Textile | R  Geochron | 2515 (375) | 1600 BC-400 AD | 0-1700 | 0-19 | 0-24 |
| 15 | Eye | R  Geochron | 1880 (95) | 60 BC-390 AD |  |  |  |

^a^ R, radiometric technique; A, AMS.

^b^ Calculated using % C Dead Sea bitumen (78%; [3]).

References

1. Clark K (2006) *Tracing the Evolution of Organic Balm use in Egyptian Mummification via Molecular and Isotopic Signatures*. PhD Thesis, University of Bristol, UK.
2. Aufderheide AC, Nissenbaum A, Cartmell LL (2004) Radiocarbon date recovery from bitumen-containing Egyptian embalming resins. *J. Soc. Study Egypt. Ant.* 31, 87-96.
3. 3. Connan J, Nissenbaum A, Dessort D (1992) Molecular Archaeology - Export of Dead-Sea Asphalt to Canaan and Egypt in the Chalcolithic - Early Bronze-Age (4^th^-3^rd^ Millennium BC). *Geochim. Cosmochim. Acta* 56, 2743-2759.
